# Methane Dynamics in a Tropical Serpentinizing Environment: The Santa Elena Ophiolite, Costa Rica

**DOI:** 10.3389/fmicb.2017.00916

**Published:** 2017-05-23

**Authors:** Melitza Crespo-Medina, Katrina I. Twing, Ricardo Sánchez-Murillo, William J. Brazelton, Thomas M. McCollom, Matthew O. Schrenk

**Affiliations:** ^1^Center for Education, Conservation and Research, Inter-American University of Puerto RicoSan Juan, PR, United States; ^2^Department of Biology, University of UtahSalt Lake City, UT, United States; ^3^Stable Isotope Research Group, School of Chemistry, National University of Costa RicaHeredia, Costa Rica; ^4^Laboratory for Atmospheric and Space Physics, Center for Astrobiology, University of Colorado BoulderBoulder, CO, United States; ^5^Department of Earth and Environmental Sciences, Michigan State UniversityEast Lansing, MI, United States

**Keywords:** tropical serpentinization, methane, methanogenesis, metagenomics, bioenergetics

## Abstract

Uplifted ultramafic rocks represent an important vector for the transfer of carbon and reducing power from the deep subsurface into the biosphere and potentially support microbial life through serpentinization. This process has a strong influence upon the production of hydrogen and methane, which can be subsequently consumed by microbial communities. The Santa Elena Ophiolite (SEO) on the northwestern Pacific coast of Costa Rica comprises ~250 km^2^ of ultramafic rocks and mafic associations. The climatic conditions, consisting of strongly contrasting wet and dry seasons, make the SEO a unique hydrogeological setting, where water-rock reactions are enhanced by large storm events (up to 200 mm in a single storm). Previous work on hyperalkaline spring fluids collected within the SEO has identified the presence of microorganisms potentially involved in hydrogen, methane, and methanol oxidation (such as *Hydrogenophaga, Methylobacterium*, and *Methylibium* spp., respectively), as well as the presence of methanogenic Archaea (such as *Methanobacterium*). Similar organisms have also been documented at other serpentinizing sites, however their functions have not been confirmed. SEO's hyperalkaline springs have elevated methane concentrations, ranging from 145 to 900 μM, in comparison to the background concentrations (<0.3 μM). The presence and potential activity of microorganisms involved in methane cycling in serpentinization-influenced fluids from different sites within the SEO were investigated using molecular, geochemical, and modeling approaches. These results were combined to elucidate the bioenergetically favorable methane production and/or oxidation reactions in this tropical serpentinizing environment. The hyperalkaline springs at SEO contain a greater proportion of Archaea and methanogens than has been detected in any terrestrial serpentinizing system. Archaea involved in methanogenesis and anaerobic methane oxidation accounted from 40 to 90% of total archaeal sequences. Genes involved in methanogenic metabolisms were detected from the metagenome of one of the alkaline springs. Methanogenic activities are likely to be facilitated by the movement of nutrients, including dissolved inorganic carbon (DIC), from surface water and their infiltration into serpentinizing groundwater. These data provide new insight into methane cycle in tropical serpentinizing environments.

## Introduction

Serpentinization is the aqueous alteration of low-silica ultramafic rocks, mainly olivine and pyroxenes, into serpentinite, brucite, magnetite, and other minerals (Moody, 1976). This reaction produces hydrogen gas (H_2_) and the reducing conditions that favor the abiogenic synthesis of methane and higher molecular weight hydrocarbons through Fischer-Tropsch-type reactions (e.g., McCollom and Seewald, 2007; Proskurowski et al., 2008). Serpentinization also produces favorable conditions for the potential biogenic formation of methane through the activity of chemolithoautotrophic microorganisms, however the occurrence and relative importance of abiogenic vs. biogenic methanogenesis processes is enigmatic (Wang et al., 2015; Etiope, 2016; Kohl et al., 2016; Miller et al., 2016).

The relationship between serpentinization and life has been relatively well studied in submarine hydrothermal systems (Schrenk et al., 2004; Kelley et al., 2005; Brazelton et al., 2006; Quéméneur et al., 2014). The study of the microbiology and geochemistry of terrestrial serpentinites has accelerated in the past several years at numerous locations globally, where meteoric water infiltrates and interacts with obducted ultramafic rock and mixes with serpentinizing fluids. Such settings have been described in the Sultanate of Oman (Barnes and O'Neil, 1978; Bath et al., 1987; Miller et al., 2016), Italy (Cipolli et al., 2004), Portugal (Marques et al., 2008; Tiago and Veríssimo, 2013), Spain (Etiope et al., 2016), Canada (Brazelton et al., 2013; Szponar et al., 2013), the Philippines (Abrajano et al., 1990; Cardace et al., 2015; Woycheese et al., 2015), and California (Barnes et al., 1967; Cardace et al., 2013; Morrill et al., 2013; Suzuki et al., 2013), among other locations.

**Figure 1:**
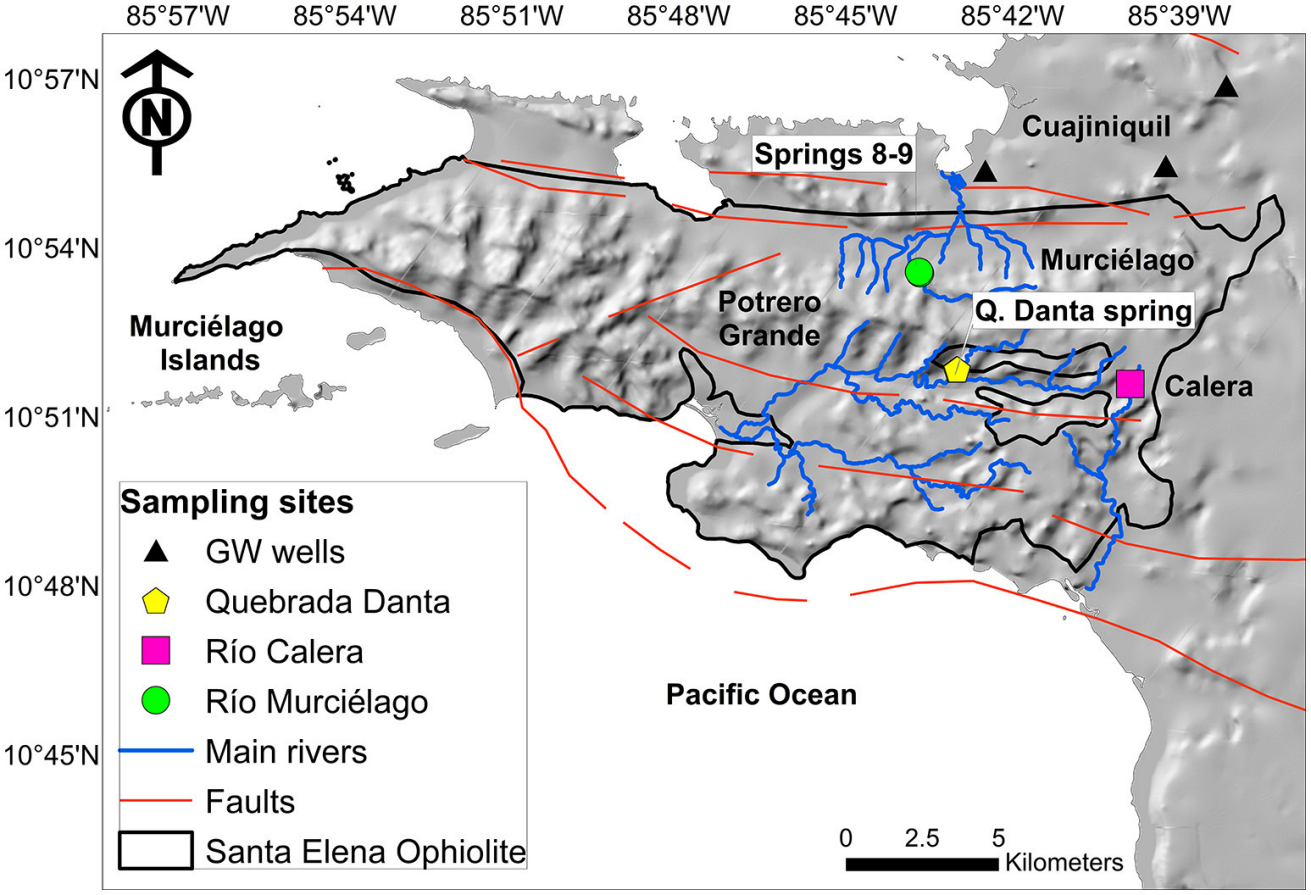
**Map of the study area**. The bold-black line denotes the Santa Elena Ophiolite boundary, dominated by ultramafic mantle rocks based on Gazel et al. (2006). Sample locations are color-coded: alkaline spring systems within Murciélago and Potrero Grande watersheds are identified by green and yellow symbols, respectively; black triangles denote groundwater wells; pink square stands for a control surface water site. Blue and orange lines are the main perennial rivers and faults, respectively.

More recently, a terrestrial serpentinization site was described in a tropical setting at the Santa Elena Ophiolite (SEO), which comprises over 250 km^2^ of ultramafic rocks and mafic associations along the northwestern Pacific coast of Costa Rica (Sánchez-Murillo et al., 2014; Figure 1). This system is characterized by warm air temperature (up to 38°C during the dry season months) and extreme variation in precipitation conditions between wet (May–November) and dry (December–April) seasons, resulting in a distinctive hydrogeological setting (Sánchez-Murillo et al., 2014). These dynamic hydroclimatic conditions make the hyperalkaline springs at SEO unique, and potentially enhance rock weathering processes and the delivery of nutrients and oxidants likely stimulating subsurface microbial activity.

Microorganisms involved in the methane cycle are abundant in marine hydrothermal serpentinizing systems (Brazelton et al., 2006). At terrestrial serpentinization sites, however, even though there is abundant methane (Wang et al., 2015), there is less evidence of the presence and activity of methanogenic and methanotrophic microorganisms. Sequences related to the anaerobic methanotrophic archaea ANME-1a have been previously detected from the springs in Cabeço de Vide aquifer in Portugal (Tiago and Veríssimo, 2013). Methanogenic taxa have been detected in springs from the Voltri Massif, Italy (Brazelton et al., 2017) and wells from the Samail Ophiolite in the Sultanate of Oman (Miller et al., 2016) and from carbonate samples collected from serpentinizing seeps in Manleluag Spring National Park, Philippines (Woycheese et al., 2015). Microcosm studies using ^13^C-labeled substrates demonstrated that native microbial communities from The Cedars (California, USA) spring water and sediments were capable of methanogenesis and acetogenesis (Kohl et al., 2016). Previous work in SEO detected the presence of microorganisms involved in the methane cycle, such as methanotrophic bacteria from the families *Methylococcaceae, Methylobacteriaceae*, and *Methylocystaceae*, and methanogenic archaea from the orders *Methanobacteriales, Methanocellales*, and *Methanomicrobiales* (Sánchez-Murillo et al., 2014). Here we investigate in more detail the presence of these organisms and their metabolic potential in fluids collected from different sites within this tropical serpentinizing setting, using molecular, geochemical, and modeling approaches. The results are discussed in light of the unique precipitation and hydrological processes within the SEO.

### Hydroclimatic conditions at SEO

Four regional air circulation processes predominantly control the climate of the SEO: NE trade winds, the latitudinal migration of the Intertropical Convergence Zone (ITCZ), cold continental outbreaks, and sporadic influence of tropical cyclones (Waylen et al., 1996; Sáenz and Durán-Quesada, 2015). These circulation processes produce two predominant rainfall maxima, one in May and June and the second one in August-September-October, which are interrupted by a relative minimum in July known as the Mid-Summer Drought (Magana et al., 1999; Maldonado et al., 2013). In addition to these circulation processes, the continental divide (i.e., a mountainous range that extends from NW to SE) also influences precipitation patterns across the country, dividing the territory into the Caribbean and Pacific slopes. The SEO (located on the northwestern Pacific slope) receives on average 1,464 mm/year of rainfall (based on 10 years of historical records at the Santa Rosa climatological station, Sánchez-Murillo et al., 2014). This region is highly dependent on the variations of the El Niño/Southern Oscillation cycles. For instance, La Niña (cold and wet phase) produces a considerable rainfall increase up to 3,000 mm, while El Niño (warm and dry phase) years are characterized by annual precipitation below 1,200 mm and a dry period usually covering 5–7 months. Seasonal temperature variation is low; mean annual maximum and minimum ambient temperatures are 31 and 23°C, respectively. During the dry period, maximum temperatures can reach up to 38°C (Sánchez-Murillo et al., 2014). The northwestern Pacific region of Costa Rica has estimated regional groundwater recharge rates of < 300 mm/year (Mulligan and Burke, 2005). Baseflow recession starts in November and reaches its minimum in late April. Most of the groundwater recharge in the SEO occurs between May and October. Commonly, terrestrial serpentinization and alkaline spring studies have been conducted in temperate regions (e.g., Tablelands, Canada; California Coast Range, USA; Gruppo di Voltri, Italy; Othrys, Greece) and at subtropical sites (e.g., Samail, Oman). In these regions precipitation is mostly composed of snow and rainfall events, meteoric recharge occurs in a short time during spring runoff or in relatively slow snowmelt rates. Coastal temperate sites might experience intermittent rainfall at relatively low intensities. Furthermore, in arid and semi-arid areas where precipitation events are scarce and isolated, infiltration is limited due to large evaporation losses. Contrary to the previous scenarios, in tropical environments like the SEO (Sánchez-Murillo et al., 2015), the Zambales Ophiolite and the Palawan Ophiolite (Abrajano et al., 1988; Cardace et al., 2015; Woycheese et al., 2015) precipitation amounts and rainfall intensities are usually greater and occur throughout several months, which facilitate infiltration and deep percolation, and thus, water-rock reactions, may be enhanced. The SEO differs from the Philippines serpentinites in that rainfall and recharge is punctuated into a short (few-month) interval.

## Materials and methods

### Site description and sample collection

During a field sampling campaign in February 2014 (dry season), fluid and gas samples were collected from three hyperalkaline springs: Spring 9 and Spring 8, located at the Murciélago river watershed, and Quebrada Danta (Q. Danta) located in the Potrero Grande watershed (Figure 1). Upstream locations to the springs were sampled (Murciélago Upstream and Q. Danta Upstream, respectively) for comparison purposes. Three private and municipal wells located nearby the SEO were also sampled (30–70 m depth): Pozo Murciélago (P. Murciélago), Pozo Nuevo (P. Nuevo), and Pozo Aguas Calientes (P. Aguas Calientes), as well as one control location at Río Calera (R. Calera) (Figure 1). Although the groundwater wells are located outside SEO, their recharge and flow paths originate within the ophiolite complex (Sánchez-Murillo et al., 2014).

Potrero Grande watershed (Figure 1) is characterized by a 10.3 km long floodplain with very steep tributaries (~33% slope), such as Q. Danta. Active erosion processes resulted in greater peridotite exposure among all watersheds in the SEO. Vegetation (i.e., deciduous trees) is mostly located in riparian areas, whereas tropical dry forest grass is common on the upper part of the stream canyons. Carbon and nutrient contributions from the topsoil layer are negligible during baseflow periods, and it solely represents subsurface conditions. The hyperalkaline system (Q. Danta) within Potrero Grande is located in the headwaters about 121 m a.s.l. Murciélago River is characterized by a relatively narrow valley and a greater presence of riparian vegetation. The hyperalkaline springs within the Murciélago watershed are located at the bottom portion of the catchment (78 m a.s.l.). Overall, field evidence suggests that hyperalkaline seepages are numerous and are active late in the baseflow period of perennial streams (December to April). Hyperalkaline springs often form shallow pools characterized by moderate turbidity, thin white films of carbonate precipitates, and extensive yellow-brown carbonate deposits.

Surface waters were exclusively collected at the flowing sections of streams to avoid stagnant ponds with biased evaporative signals (Sánchez-Murillo and Birkel, 2016). Groundwater samples were collected using Teflon sterile and disposable geobailers (Geotech Environmental Equipment, Colorado, USA) and automated pumping (P. Murciélago). Spring samples were collected according to following criteria: evidence of continuous water flow from the rock, close to bubbling zones, and near the most reductive point. The sampling campaign was designed to target baseflow conditions. Baseflow is described as the cumulative outflow from all upstream riparian aquifers during rainless periods (Brutsaert, 2005), therefore, it represents the most average geochemical characteristics of any particular watershed or aquifer (Sánchez-Murillo et al., 2015).

Fluid and gas samples were collected from all sites following methods in the subsections below. Standard physicochemical measurements (i.e., water temperature, pH, Eh, electrical conductivity (EC), total dissolved solids, and salinity) were recorded using handheld probes (Oakton PC Testr 35 and Oakton Testr 10), which were calibrated twice a day using standard solutions.

### Gas and aqueous geochemical characterization

Water samples for dissolved methane concentration and isotopic composition analyses were collected from the spring source using a bubble-free 60 mL syringe with Tygon tubing attached. The samples were carefully transferred to a He-purged 160 mL serum bottle and crimp sealed immediately. For H_2_, a 10 mL sample was collected following the same protocol and transferred to a 14 mL serum vial. For dissolved inorganic carbon (DIC), a 14 mL serum vial, containing one NaOH pellet, was filled to the top, capped with a butyl rubber stopper, and microbial activity was arrested using saturated ZnCl_2_ (80 μM final conc.). All samples were collected in triplicate and were sent for analysis as a contracted service to JBL Analytical Services (http://www.joyeresearchgroup.uga.edu; University of Georgia, GA, USA).

The filtrate from the 0.2 μm Sterivex filter cartridges (EMD Millipore, Billerica, MA) used for molecular sampling (see methods below) was collected into an acid-cleaned 30 mL Nalgene bottle (Tupas et al., 1994; Joye et al., 2010) for dissolved organic carbon (DOC) and nitrogen and phosphorous species: total dissolved nitrogen (TDN), ammonium (${\text{NH}}_{4}^{+}$), nitrite (${\text{NO}}_{2}^{-}$), nitrite + nitrate (${\text{NO}}_{2}^{-}$ + ${\text{NO}}_{3}^{-}$), total dissolved phosphorous (TDP), and orthophosphate (${\text{PO}}_{4}^{-3}$). Samples were transported in cold bags from the field and were frozen at −20°C immediately upon arrival to the field station (2–6 h after sampling). Samples were sent for analysis to JBL Analytical Services (University of Georgia, GA, USA).

Samples for water stable isotope analysis were collected in 30 mL glass E-C borosilicate bottles with tetrafluoroethylene (TFE)-lined caps (Wheaton Science Products, USA). Bottles were filled completely, leaving no head space, covered with parafilm (Thermo Scientific, USA) avoiding exchange with atmospheric moisture, and stored upside down at 5°C until analysis. Water stable isotope analyses were conducted at the Stable Isotope Research Group facilities of the National University (Heredia, Costa Rica) using a Cavity Ring Down Spectroscopy (CRDS) water isotope analyzer L2120-*i* (Picarro, USA). The secondary standards were: Moscow Tap Water, MTW (δ^2^H = −131.4‰, δ^18^O = −17.0‰), Deep Ocean Water, DOW (δ^2^H = −1.7‰, δ^18^O = −0.2‰), and Commercial Bottled Water, CAS (δ^2^H = −64.3‰, δ^18^O = −8.3‰). MTW and DOW standards were used to normalize the results to the VSMOW2-SLAP2 scale, while CAS was used as a quality control and drift control standard. The analytical long-term precision was: ±0.5‰ (1 s) for δ^2^H and ± 0.1‰ (1 s) for δ^18^O.

### Cell counts

Fluids were preserved in 3.7% (v/v) formaldehyde (final concentration) for enumeration of cell abundances and stored at 4°C until processing. The preserved fluids were filtered through a 0.2 μm black polycarbonate filters (Whatman; PA, USA), and cells were stained with 1 μg/ml of 4′,6-diamidino-2-phenylindole (DAPI) and counted by epifluorescence microscopy using appropriate filter sets according to previously published protocols (Hobbie et al., 1977; Schrenk et al., 2003).

### DNA extraction

For molecular characterization of the microbial communities, between one to three liters of fluids were filtered in the field though 0.22 μm Sterivex™ –GV filter units (EMD Millipore, MA, USA) using a Masterflex® E/S portable sampler (Cole-Parmer, IL, USA). Filters were covered with ice during the filtration process, then capped and flash frozen in liquid nitrogen, and stored at −80°C until processing. Extraction of total genomic DNA followed previously described protocols (Huber et al., 2002; Sogin et al., 2006). DNA extracts were purified with the DNA Clean and Concentrator™-5 kit (Zymo Research, CA, USA) according to the manufacturer's instructions. DNA extracts were quantified with a Qubit® dsDNA High Sensitivity Assay kit in a Qubit® 2.0 Fluorometer (Life Technologies, NY, USA).

### 16S rRNA gene enumeration

Quantification of 16S rRNA gene copies were determined by quantitative polymerase chain reaction (q-PCR) on a BioRad C-1000 thermo-cycler with a q-PCR module using the SsoAdvanced SybrGreen Assay (BioRad; CA, USA). Domain-specific primers, 958F and 1048R for Archaea and 967F and 1064R for Bacteria, targeted the V6 hypervariable region of the 16S rRNA gene (Sogin et al., 2006). Gene copy numbers were calculated using standard curves generated by amplification of DNA from *Methanocaldococcus jannaschii* for Archaea and *Escherichia coli* for Bacteria. Amplification efficiencies were 70% for the Archaeal and 93% for the Bacteria qPCR reactions, respectively.

### 16S rRNA gene tag sequencing and data analysis

Purified DNA was submitted to the Josephine Bay Paul Center, Marine Biological Laboratory (http://www.mbl.edu/jbpc/) for amplicon sequencing of archaeal and bacterial 16S rRNA genes with an Illumina MiSeq instrument with domain-specific primers targeting the V4-V5 hypervariable regions of the 16S rRNA gene (518F-926R for bacteria and 517F-958R for archaea), following the methods described by Nelson et al. (2014). Quality-filtering of the sequences was conducted via the Visualization and Analysis of Microbial Population Structures (VAMPS) pipeline (Huse et al., 2014). These data are publicly available at https://vamps.mbl.edu/ under the project code DCO_BRZ and the sample code Serp_SEO as well as the NCBI SRA database under SRP096759 and Bioproject accession number PRJNA361138.

The 16S rRNA gene amplicon sequences were processed as described previously by Brazelton et al. (2017). Briefly, further quality-control of the sequences was completed in mothur (v. 1.36.1; Schloss et al., 2009) to remove sequences with homopolymers >9 and ambiguous bases >0. The mothur command pre.cluster (diff = 1) was run, reducing the number of unique samples from 1,531,396 to 1,044,544 for Bacteria and 862,835 to 616,217 for Archaea. The resulting unique sequences were considered operational taxonomic units (OTUs) for this study. Taxonomic classification of all OTUs was performed with mothur using the SILVA reference alignment (SSURef v.123.1) and taxonomy outline (Pruesse et al., 2007).

This current study focuses on the methane cycling members within the bacterial and archaeal communities. To assess the abundance of methanotrophic bacteria, the following genera were searched within the taxonomic dataset: *Methylococcus, Methylocaldum, Methylohalobius, Methyothermus, Methylobacter, Methylomicrobium, Methylomonas, Methylosarcina, Methylosoma, Methylosphaera, Crenothrix*, and *Clonothrix* of the family *Methylococcacaeae* within the Gammaproteobacteria; *Methylocystis* and *Methylosinus* of the family Methylocystaceae and *Methylocella* and *Methylocapsa* of the family Beijerinckiaceae within the Alphaproteobacteria (Hanson and Hanson, 1996; Op den Camp et al., 2009 and the references therein). Additionally, the members of the genus *Methylacidiphilum* within phylum Verrucomicrobia (Op den Camp et al., 2009) and the Ca. genus Methylomirabilis of the candidate phylum NC10 (Ettwig et al., 2010) were also searched for within the bacterial taxonomic dataset. Archaea classified as belonging to Anaerobic Methane Oxidizing Archaea (ANME) were identified as potential methanotrophs. For methanogenic archaea, sequences taxonomically classified as belonging to the orders Methanopyrales, Methanococcales, Methanobacteriales, Methanomicrobiales, Methanocellales (Sakai et al., 2008), Methanoplasmatales (Paul et al., 2012), and Methanosarcinales (Thauer et al., 2008) were identified. The relative abundances of these taxa were calculated with the get.relabund command in mothur.

### Metagenomic sequencing and data analysis

Purified DNA from two samples (Spring 9 and Murciélago Upstream) was submitted to the Josephine Bay Paul Center, Marine Biological Laboratory for shotgun metagenomic sequencing. Metagenomic libraries were constructed with the Nugen Ultralow Ovation kit according to the manufacturer's instructions. Paired-end sequencing with a 100 cycle Illumina HiSeq run generated partial ~30 bp overlaps, and six libraries were multiplexed per lane. The raw metagenomic sequence data are publically available in the NCBI SRA database under the BioProject accession number PRJNA340462.

Quality control of the metagenomic reads was performed to remove low quality and sequencing artifact reads. Cutadapt v.1.9 (Martin, 2011) was used to identify and remove reads with Illumina adapters at the 5′-end of the sequence and to trim Illumina adapters at the 3′-end of the sequence. Identical and 5′-prefix replicates were also removed (Gomez-Alvarez et al., 2009). Nucleotides (0–3) at the beginning and end of reads were cropped from all reads in that sample if those positions exhibited nucleotide frequencies inconsistent with the nucleotide frequency distribution for the rest of the read. Low-quality bases were removed from the ends of the reads, and the remaining sequence was scanned 6 base pairs at a time and trimmed where the mean quality score fell below a score of 28. Reads that did not pass a minimum length threshold of 62 bp after quality and adapter trimming were removed from the dataset. Metagenomic assembly of each sample was performed by Ray Meta v.2.3.1 (Boisvert et al., 2012). A kmer of 61 was chosen after manual inspection of assemblies with kmer values of 31, 41, 51, and 61. High-quality reads from each sample were mapped onto each assembly with Bowtie2 v.2.2.6 (Langmead and Salzberg, 2012). Metagenomic assembly statistics can be found in Table S1. The Prokka pipeline (Seeman, 2014) was used for gene prediction and functional annotation, with the arguments –metagenome and –proteins in Prokka v.1.12 with gene prediction by Prodigal v.2.6.2 (Hyatt et al., 2010). The database provided was the Kyoto Encyclopedia of Genes and Genomes, release 2016-09-26 (Ogata et al., 1999). Predicted protein abundances (in units of reads per kilobase) were calculated with HTSeq v.0.6.1 (Anders et al., 2014), and the final normalized coverage was calculated by normalizing to the total number of fragments in the smallest metagenome. A summary of the methane-cycling genes and their KEGG IDs searched for within this dataset can be found in Table S2.

### Bioenergetics calculations

To assess the amount of chemical energy potentially available to support chemolithoautotrophic organisms at the study sites, a series of calculations were performed to estimate the Gibbs energy of several metabolic reactions based on the measured fluid compositions. For the calculations, fluid speciation calculations were first performed with Geochemist's Workbench (Aqueous Solutions LLC, Champaign, IL) to estimate activities of dissolved species involved in the metabolic reactions, such as dissolved CO_2_. The default thermo.com.V8.R6+.tdat database supplied with the program was used for the calculations. Since data were available for only some components of the fluid, nominal amounts of Na and Cl (10–15 mM for Na and Cl) were included in the calculations to account for the measured salinity and achieve charge balance since these are typically the major dissolved ion components at sites of terrestrial serpentinization (e.g., Morrill et al., 2013).

The amount of energy available from several potential metabolic reactions involving methane (Tables S3, S4) was then calculated according to the familiar equation:

(1) $$\begin{array}{c} \Delta \text{G}\;=\;\Delta {\text{G}}^{{}^{\circ}}\;+\text{ RT ln}\;Q \end{array}$$

where ΔG is the Gibbs energy of reaction (J/mol), ΔG° the standard Gibbs energy, R the universal gas constant (J/mole K), T the temperature (K), and *Q* the activity quotient of the compounds involved in the reaction. The latter factor, *Q*, takes into account the contribution of the fluid composition to the Gibbs energy of each reaction, and was calculated using the activities determined by the fluid speciation models. Values of the ΔG° were calculated using SUPCRT92 (Johnson et al., 1992) with the default database. Since measurements of dissolved O_2_ were not available, calculations for aerobic methanotrophy were performed assuming concentrations equivalent to 0.1 and 1.0% of saturation with respect to air (Table S3). Similarly, calculations for acetoclastic methanogenesis assume acetate accounts for 1 or 10% of measured DOC concentrations (Table S4), which are within the range previously reported for the Coast Range Ophiolite (Crespo-Medina et al., 2014). To better facilitate comparisons between different metabolic pathways, the total amount of energy available from each of these reactions per liter of fluid was estimated by multiplying the Gibbs energy by the concentrations of the reaction components in the fluids, taking into account which of the reactants was the limiting component (e.g., McCollom and Shock, 1997).

## Results

### Aqueous, volatile, and stable isotope geochemistry

Fluids from SEO's springs are highly alkaline with pH ranging between 11.2 and 11.6 and highly reducing, with an oxidation-reduction potential (ORP) ranging from −380 to −251 mV. Overall, surface water from upstream sites and groundwater in proximity of SEO are relatively alkaline with pH values ranging from 7.54 up to 8.90. Upstream sites and groundwater wells exhibited oxidizing conditions with ORP values ranging from +66 up to +97 mV. These conditions are consistent with springs sampled at other terrestrial serpentinization sites (Schrenk et al., 2013, and reference therein). The fluid temperature ranged from 26.1° to 29.2°C, with the temperature at the springs measuring 1–3°C greater than the respective upstream counterparts (Table 1). Electrical conductivity (EC) ranged from 470 up to 700 μS/cm. EC values upstream from the hyperalkaline springs were consistently lower, while groundwater wells presented the greatest EC values. The DOC concentration in the spring fluids ranged from 5.6 to 73.2 μM (Table 1), while upstream sites ranged from 67.1 to 103.0 μM. Groundwater wells presented low DOC values ranging from 11.6 up to 25.9 μM. The fluids are enriched in nitrogen and phosphorous species compared to other terrestrial serpentinizing sites (Tiago et al., 2004; Morrill et al., 2013; Cardace et al., 2015), though to a lesser extent than was previously reported for fluids from the Coast Range Ophiolite in California (Crespo-Medina et al., 2014). Both DOC and nutrients (nitrogen and phosphorous species) were slightly elevated at Spring 8 and Spring 9 compared to Q. Danta (Table 1). Hyperalkaline springs and groundwater wells presented a nearly uniform δ^18^O (−6.65 to −7.22‰) and δ^2^H (−45.1 to −50.4‰) composition. The sampling site upstream from Q. Danta spring was more enriched (δ^18^O = −4.33‰and δ^2^H = −33.4‰).

**Table 1:** Physicochemical, geochemical, and isotopic analysis of samples collected from Santa Elena Ophiolite^*^.

**Sample name**	**Q. Danta**	**Q. Danta Upstream**	**Spring 8**	**Spring 9**	**Murciélago Upstream**	**R. Calera**	**P. Murciélago**	**P. Nuevo**	**P. Aguas Calientes**
Sample type	Spring	River	Spring	Spring	River	River	Well	Well	Well
**PHYSICOCHEMICAL PARAMETERS**									
pH	11.59	8.42	11.54	11.54	8.9	8.61	7.54	8.3	7.26
Temp. (°C)	29.2	27.9	26.1	26.4	24	25.3	28	30.2	30.2
Conductivity (μS/cm)	542	470	543	466	492	493	700	696	603
TDS (ppm)	358	334	386	333	306	349	498	535	428
Salinity (ppm)	412	357	414	351	326	372	535	496	459
ORP (mV)	−251	66	−331	−348	35	148	97	76	70
**AVERAGE GEOCHEMICAL PARAMETERS (μM)**									
DOC	5.6	103.0	73.2	41.2	67.1	59.5	11.6	20.2	25.9
DIC (μM)	126.3	597.3	227.0	254.3	533.7	663.3	748.0	417.7	644.3
δ^13^C-CO_2_ (‰)	−20.1	−17.9	−17.9	−20.8	−19.8	−18.6	−19.5	−18.6	−18.7
TDN	0.4	6.6	14.2	1.7	1.7	2.6	62.4	3.5	39.2
NOx	0.8	2.5	0.3	0.2	0.3	1.8	58.3	0.2	36.8
NO_2_	0.0	0.1	0.0	0.0	0.1	0.0	0.0	0.0	0.0
${\text{NH}}_{4}^{+}$	1.3	0.6	1.7	0.8	0.1	0.1	2.1	4.1	1.5
DON	bdl^**^^a^	3.5	12.2	1.1	1.3	0.7	2.0	bdl	0.9
${\text{PO}}_{4}^{3-}$	0.1	1.6	0.2	0.3	1.6	1.9	2.7	1.7	2.7
TDP	0.1	0.2	0.1	0.1	0.2	0.2	0.4	0.6	1.1
**AVERAGE GASEOUS GEOCHEMISTRY AND ISOTOPIC COMPOSITION**									
CH_4_ (μM)	145.0	0.3	870.7	912.3	14.3	0.3	0.3	0.3	0.2
δ^13^C-CH_4_ (‰)	−44.0	bdl^b^	−0.9	−2.2	1.3	bdl	bdl	bdl	bdl
H_2_ (μM)	38.3	9.2	10.9	53.1	0.8	23.7	1.0	0.8	1.0
**WATER ISOTOPES**									
δ^18^O (‰)	−7.18	−4.33	−7.22	−7.18	−7.02	−6.65	−7.12	−6.84	−6.65
δ^2^H (‰)	−50.0	−33.4	−50.4	−50.4	−45.7	−45.1	−47.6	−48.9	−45.1

* *Complete data set is presented in Table S5*.

** *bdl, below detection level*.

a *Limit of detection for DON was 0.1 μM*.

b *Samples with methane concentration of 0.3 μM or less were within the limit of the detection of the instrument, but did not give a reliable δ^13^C-CH_4_ signature (measurements with high standard deviation) and thus were not taken into consideration in this analysis*.

**Figure 2:**
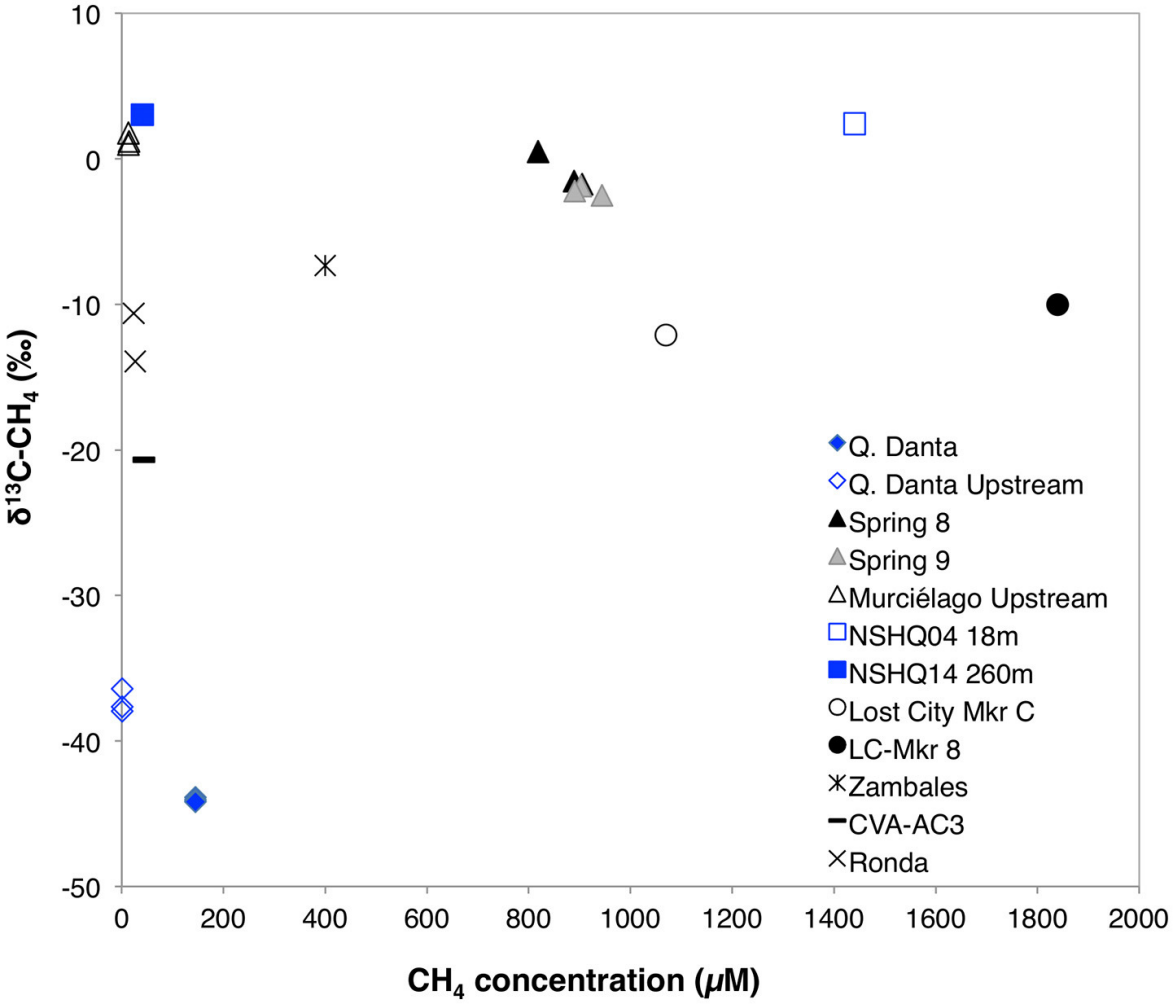
**Concentration vs. stable isotopic signature for dissolved methane in fluids from SEO in comparison with other serpentinizing fluids**. Samail Ophiolite data (red squares) was obtained from Miller et al. (2016) and Etiope (2016); Lost City data (circles) was obtained from Proskurowski et al. (2008); Zambales Ophiolite data (blue cross) was obtained from Cardace et al. (2015); and Cabeço de Vide data (black asterisk) was obtaind from Etiope et al. (2013).

Methane concentrations in SEO's spring fluids ranged from 145 to 912 μM, which is in the same range as previously described for the Coast Range Ophiolite (CA, USA) (210–1832 μM, Crespo-Medina et al., 2014), serpentinite springs at Voltri Massif (155–733 μM; Brazelton et al., 2017), and at tropical springs from Zambales and Palawan Ophiolites in Philippines 0–400 μM; Cardace et al., 2015), but slightly elevated when compared to springs at Tablelands (20.0–26.2 μM; Szponar et al., 2013). Methane concentrations were 0.3 μM at the Q. Danta upstream and 14.3 μM at the Murciélago upstream sites (Table 1). However, it is important to highlight that several bubbling sites within the Murciélago River's upstream channel were observed, which may have contributed to the methane concentrations measured in this stream. Methane concentrations in the control stream (R. Calera) and nearby groundwater wells were consistently below 0.3 μM. Hydrogen concentrations at the springs were elevated, ranging from 10.9 up to 53.1 μM, which is greater than previously described for the California site (CROMO, 0.2–0.7 μM; Crespo-Medina et al., 2014), and consistent with what has been previously observed at Voltri Massif (0.5–26.8 μM; Brazelton et al., 2017), but lower to that reported from Tablelands (585–747 μM; Szponar et al., 2013), and from Zambales Ophiolite (0–495.5 μM; Cardace et al., 2015). Spring 9 had the greatest methane and hydrogen concentrations (Table 1). Groundwater wells presented lower hydrogen concentrations (< 0.8 μM). The spring samples contain lower DIC concentration (ranging between 126 and 254 μM) than the background samples (417–748 μM). Even though DIC at the SEO springs is greater than at most terrestrial serpentinizing sites; e.g. in the Voltri Massif springs in Italy, DIC ranged from 7.8 to 29 μM (Brazelton et al., 2017), at The Cedars' springs it ranged from 6 to 70 μM (Morrill et al., 2013), while at CROMO, it ranged from 21 to 63 μM in fluids collected from established wells and 194–210 μM in a newly-drilled well (Crespo-Medina et al., 2014), the levels are 2–15 times lower than what was previously reported form another tropical serpentinizing site in Phillipines (250–3216 μM; Cardace et al., 2015). Samples with greater methane concentrations (Springs 8 and 9 within Murciélago river) were enriched in ^13^CH_4_ (Table 1, Figure 2), relative to a spring with moderate methane concentrations (Q. Danta) (Table 1, Figure 2). A detailed table with the geochemistry results from individual replicates is presented as a supplementary material (Table S5).

### DNA yields and cell abundances

In general, the hyperalkaline springs had slightly lower biomass than their respective upstream samples, as suggested from the DNA yield (ng/L) and cell count data (cells/ml) (Table 2), and slightly higher abundances than neutral pH groundwater wells. Conversely, the wells had the highest average abundances of archaeal amplicons, followed by the springs, and the upstream “background” samples (Table 2).

**Table 2:** **Microbial biomass abundance data for samples collected from Santa Elena Ophiolite**.

**Sample name (replicate)**	**DNA yield (ng/L)**	**Archaeal 16S copies per mL of sample**	**Bacterial 16S copies per mL of sample**	**Cell counts (cells/mL)**
Q. Danta (1)	34.4	2.00 × 10^3^	1.05 × 10^4^	2.90 × 10^4^
Q. Danta (2)	57.7	8.24 × 10^3^	2.74 × 10^4^	4.15 × 10^4^
Q. Danta (3)	78.2	4.92 × 10^3^	2.68 × 10^4^	3.37 × 10^4^
Q. Danta Upstream (1)	767.8	2.96 × 10^4^	2.29 × 10^5^	8.64 × 10^4^
Q. Danta Upstream (2)	2111.4	3.51 × 10^4^	4.47 × 10^5^	1.94 × 10^4^
Q. Danta Upstream (3)	1004.1	2.99 × 10^1^	9.63 × 10^3^	1.63 × 10^4^
Spring 8 (1)	68.3	5.40 × 10^3^	1.66 × 10^4^	3.44 × 10^4^
Spring 8 (2)	1499.0	5.55 × 10^4^	2.71 × 10^5^	4.39 × 10^4^
Spring 8 (3)	366.6	1.74 × 10^4^	8.37 × 10^4^	7.18 × 10^4^
Spring 8 (4)	559.7	9.73 × 10^3^	6.90 × 10^4^	2.66 × 10^4^
Spring 9 (1)	501.8	3.34 × 10^4^	1.74 × 10^5^	1.90 × 10^5^
Spring 9 (2)	552.3	3.11 × 10^4^	1.63 × 10^5^	1.09 × 10^5^
Spring 9 (3)	518.3	3.74 × 10^4^	2.15 × 10^5^	1.33 × 10^5^
Murciélago Upstream (1)	972.0	5.54 × 10^4^	7.20 × 10^5^	2.29 × 10^5^
Murciélago Upstream (2)	1096.4	5.71 × 10^4^	7.55 × 10^5^	2.21 × 10^5^
Murciélago Upstream (3)	1185.1	3.58 × 10^4^	6.30 × 10^5^	7.15 × 10^4^
R. Calera (1)	439.3	2.01 × 10^4^	7.18 × 10^4^	7.76 × 10^4^
R. Calera (2)	283.4	3.46 × 10^3^	1.20 × 10^4^	5.17 × 10^4^
R. Calera (3)	224.7	3.14 × 10^3^	1.07 × 10^4^	1.14 × 10^4^
P. Murciélago (1)	97.6	1.02 × 10^4^	1.41 × 10^4^	7.49 × 10^3^
P. Murciélago (2)	11.4	1.82 × 10^3^	2.45 × 10^3^	Bdl^*^
P. Murciélago (3)	13.1	2.06 × 10^3^	2.76 × 10^3^	Bdl
P. Nuevo (1)	748.5	6.84 × 10^4^	1.55 × 10^5^	9.39 × 10^4^
P. Nuevo (2)	677.5	ND	1.57 × 10^5^	7.88 × 10^4^
P. Nuevo (3)	599.9	6.21 × 10^4^	1.58 × 10^5^	1.90 × 10^5^
P. Aguas Calientes (1)	168.5	2.07 × 10^4^	2.87 × 10^4^	2.54 × 10^4^
P. Aguas Calientes (2)	159.9	1.27 × 10^4^	2.03 × 10^4^	Bdl
P. Aguas Calientes (3)	309.1	1.79 × 10^4^	8.03 × 10^4^	4.51 × 10^4^

*ND, not determined; bdl, below detection limit*.

* *detection limit for cell counts was 6.98 × 10^3^ cells per mL*.

### Abundance of methanotrophs and methanogens from 16S rRNA gene sequencing

**Figure 3:**
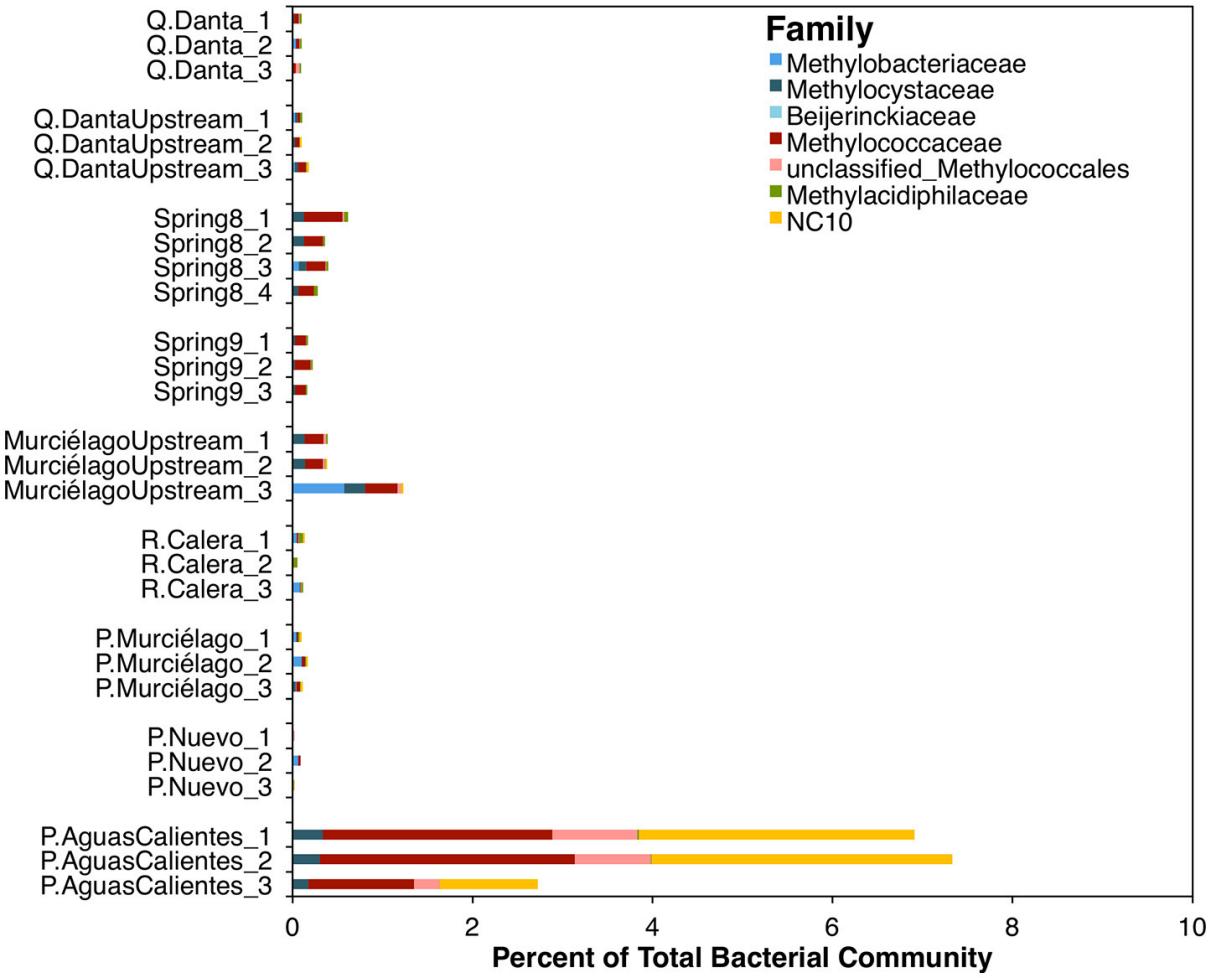
**Relative abundance of methanotrophic OTUs within the bacterial 16S rRNA amplicon dataset**.

Based upon tag sequencing of 16S rRNA marker genes, sequences related to known methanotrophic bacteria comprise < 2% of the total bacterial 16S rRNA gene sequences in all the samples, except in the fluids from P. Aguas Calientes, where sequences related to these microorganisms represent ~3–7% of the total sequences (Figure 3). From 1 to 2% of the sequences in P. Aguas Calientes belong to the Phylum NC10, whose members are capable of NO-dismutation coupled to methane oxidation (Ettwig et al., 2010).

**Figure 4:**
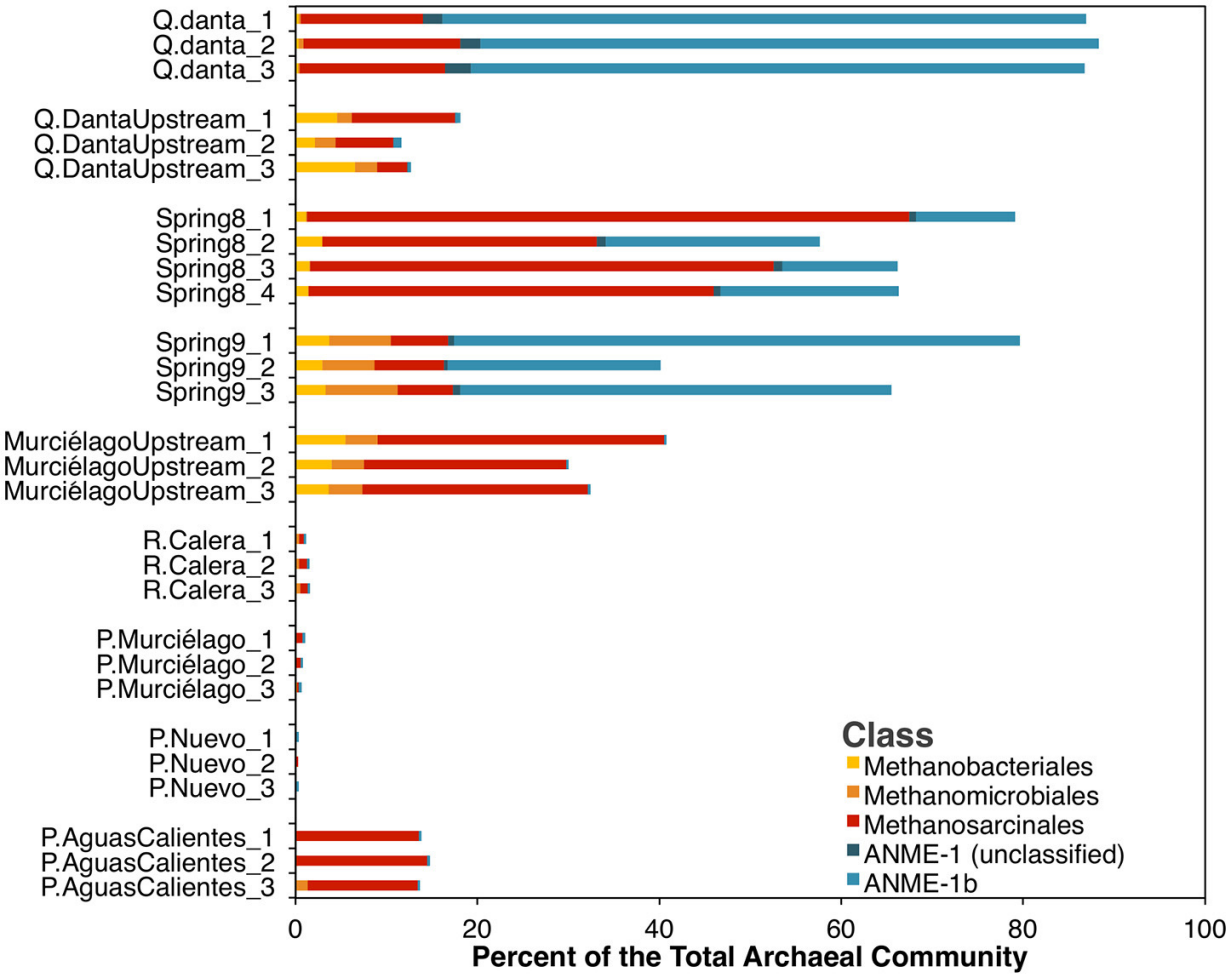
**Relative abundance of methane-cycling archaea within the archaeal 16S rRNA amplicon dataset**. Families generally associated with methanogenesis are in shades of yellow and red, while taxa generally associated with anaerobic methane-oxidation are in blue.

On the other hand, archaeal sequences related to the methane cycling (methanogenesis and AOM), accounted for ~40 to 90% of the archaeal 16S rRNA gene sequences in spring water samples, 15 to 40% in the upstream samples, and 0–15% in the control river sample (R. Calera) and in groundwater well samples (Figure 4) suggesting the importance of methane-metabolizing organisms in this system. The majority of the archaeal sequences in the Spring 8 sample belong to the order Methanosarcinales, which for the most part include methanogenic Archaea. However, it should be noted that Methanoperedens, a member of the ANME-2d clade, falls within Methanosarcinales and was detected in Spring 8 in low abundances within the 16S rRNA amplicon dataset. The majority of the sequences in Spring 9 and Q. Danta belong to the ANME-1b group, a group that is thought to represent obligate methanotrophs (Hinrichs et al., 1999).

### Metagenomic analysis of the methanogenesis pathway

**Figure 5:**
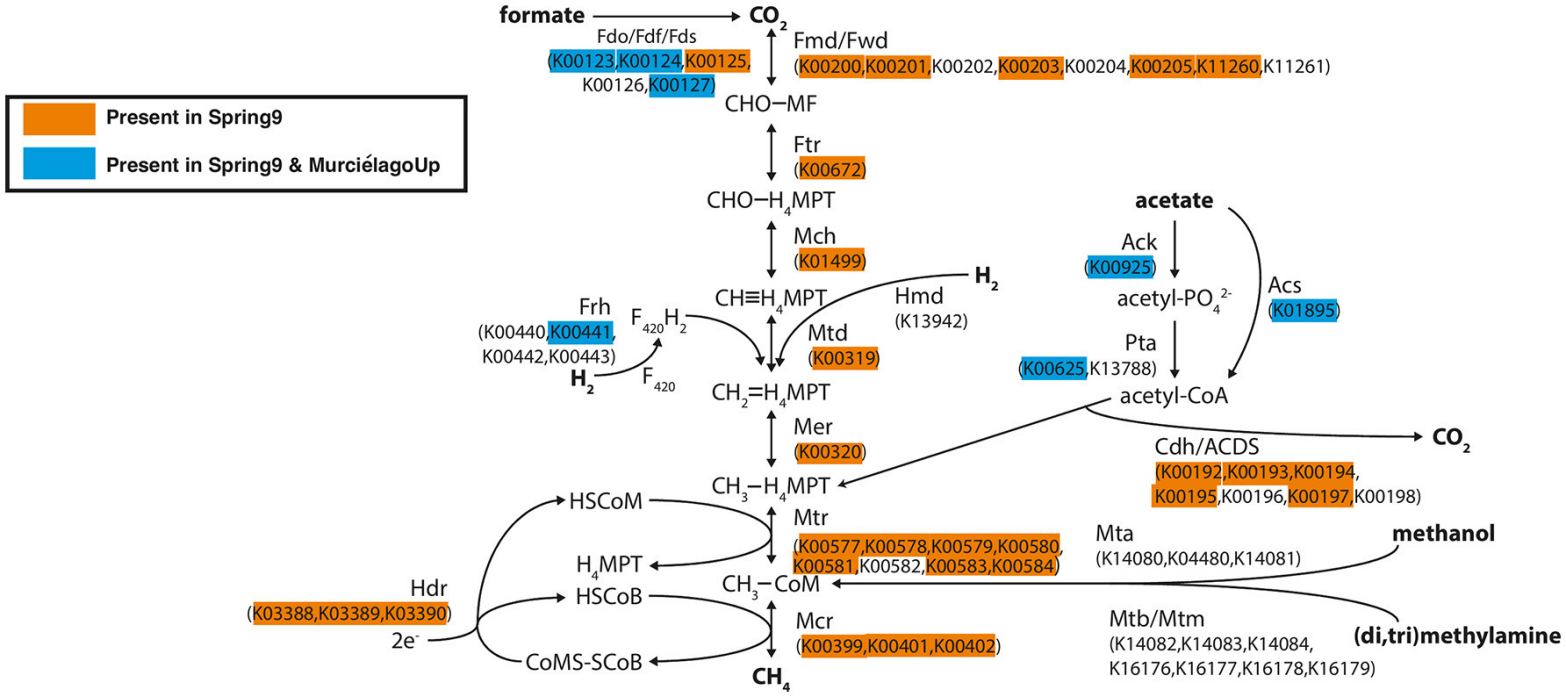
**Diagram of methanogenesis pathways from carbon dioxide, formate, acetate, methanol, and methylamines with associated protein homologs identified with KEGG IDs**. Orange-highlighted proteins are predicted to occur in the Spring 9 metagenomic assembly, and blue-highlighted proteins are predicted to occur in both the Spring 9 and Murciélago upstream assemblies. The diagram is modified from Ferry (2010).

Metagenomic sequences were obtained from Spring 9 and Murciélago Upstream, and the analysis described here focuses only on the identification of genes involved in the methane cycle (Table S2). Genes involved in aerobic methanotrophy, such as particulate methane monooxygenase (*pmoA*) or methanol dehydrogenase (*mxfA*) were not detected in the metagenomic assemblies (Table S2). However, when investigating genes involved in methanogenesis pathways (Figure 5), we were able to detect all the key genes involved in acetoclastic methanogenesis, hydrogenotrophic methanogenesis, and methanogenesis from formate in the Spring 9 metagenome. The genes for methylotrophic methanogenesis were not detected in either metagenome. The metagenome of the Murciélago Upstream sample contained only genes involved in initial steps of methanogenesis, such as those involved in the conversion of formate to CO_2_, of acetate to acetyl Co-A and for the incorporation of H_2_ into di-iron flavoprotein F_420_H_2_ oxidase.

### Bioenergetics of methane metabolism

**Figure 6:**
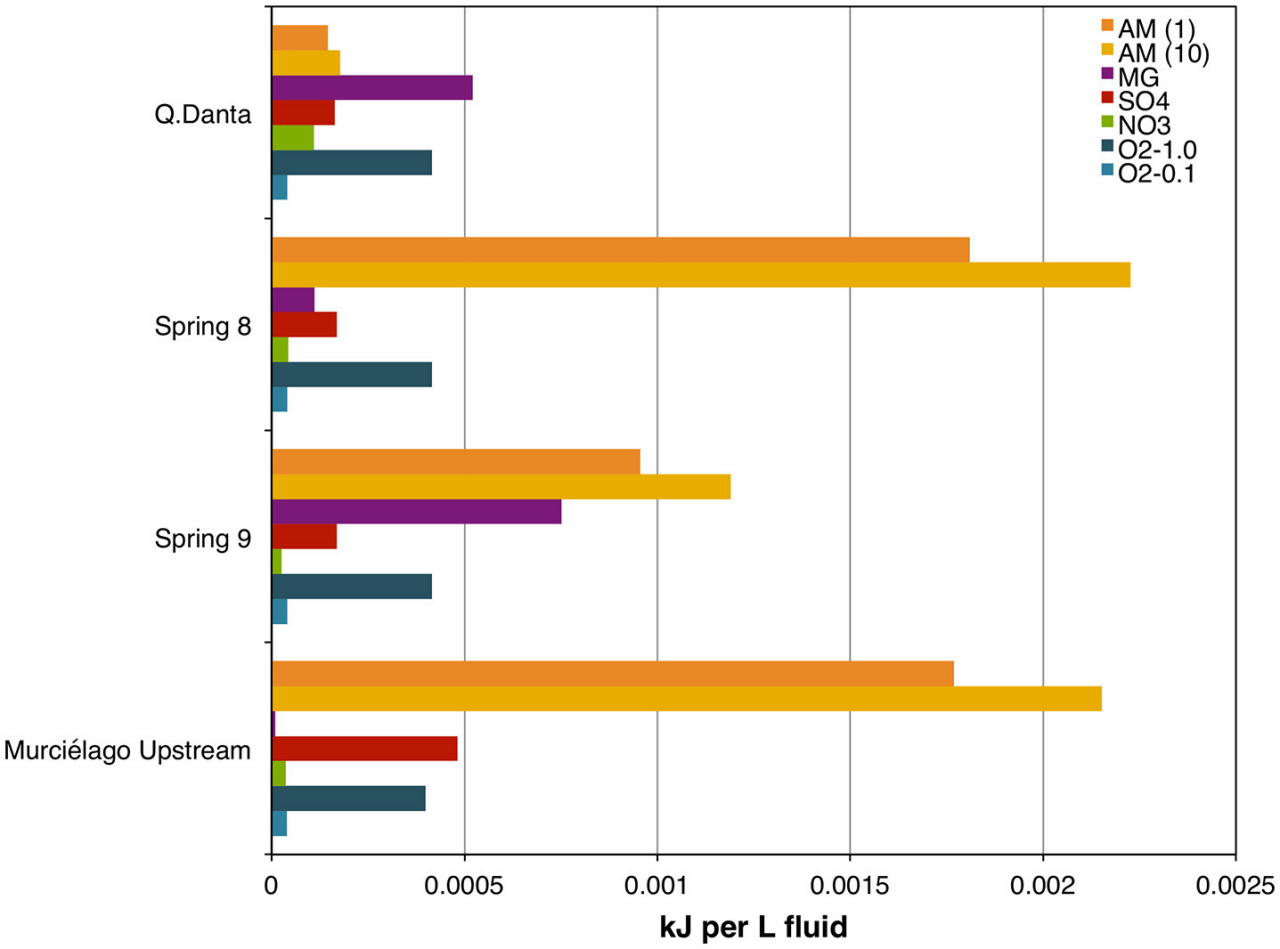
**Free energy yield (kJ per L of fluids) for methanogenic and methanotrophic processes in SEO's fluids**. AM (1), acetoclastic methanogenesis assuming 1% of DOM is acetate; AM (10), acetoclastic methanogenesis assuming 10% of DOM is acetate: MG, hydrogenotrophic methanogenesis; SO_4_, Anaerobic oxidation of methane (AOM) coupled to ${\text{SO}}_{4}^{2-}$ reduction; NO_3_, AOM coupled to ${\text{NO}}_{3}^{-1}$ reduction; O_2_-1.0, aerobic methane oxidation assuming 1% saturation with respect to air; O_2_-0.1, aerobic methane oxidation assuming 0.1% saturation with respect to air.

For Spring 8 and Spring 9, the bioenergetics calculations reflect that there is more energy (kJ per L) from acetoclastic methanogenesis than hydrogenotophic methanogenesis (although that depends, in part, on assumptions about the composition of DOM; Figure 6, Table S4), and both have greater energy yields than anaerobic methane oxidation. For Q. Danta the bioenergetics calculations reflect that there is more energy available from hydrogenotrophic methanogenesis than acetoclastic methanogenesis. Bioenergetic calculations for this system demonstrate that anaerobic methane oxidation is limited by the abundance of electron acceptors (Figure 6, Table S3).

## Discussion

As in other serpentinization-influenced ecosystems, the springs at SEO have fluids that are highly alkaline, reduced, and enriched in methane and hydrogen. The fluids at SEO had elevated concentrations of DIC and nutrients when compared to previously studied serpentinizing springs, this might be due in part by the large input of meteoric water to the system and short mean residence times. Microorganisms involved in methane cycling were detected in all three alkaline springs studied. Aerobic methanotrophic bacteria were less prevalent than ANME or methanogenic archaea in spring fluids. These results are consistent with our bioenergetics calculations, which indicate that aerobic methanotrophy is not likely to be a favorable process at depth in this system. Sequences related to ANME-1b methanotrophic archaea and to methanogens from the orders Methanosarcinales, Methanomicrobiales and Methanobacteriales accounted for 40–90% of the total archaeal sequences in all three hyperalkaline spring fluids.

The enriched δ^13^C-CH_4_ signature of the Spring 8 and Spring 9 samples suggests that a majority of methane in these samples is likely to be abiotically generated (Whiticar, 1999), although biological methanogenesis from δ^13^C-enriched substrates cannot be excluded with the current data set (Etiope, 2016). At Spring 8 and Spring 9, the biological signal of methanogenesis may be overshadowed by abiogenic contributions. On the other hand, the detection of a ^13^C depleted stable isotope signature (~ −40‰) in methane from Q. Danta may represent contributions from acetoclastic methanogenesis (Whiticar, 1999).

Bioenergetic calculations indicate that methanogenesis (acetoclastic and hydrogenotrophic) yields more energy than anaerobic methanotrophy, but the sample with the most depleted δ^13^C-CH_4_ (Q. Danta) was dominated by ANME-1b. The detection of ANME -1 archaea in methanogenic sediments from White Oak River estuary, however, suggests that these organisms are not exclusively methanotrophs and that they might be capable of methanogenesis (Lloyd et al., 2011). Alternatively, the isotopic signature may represent a combination of hydrogenotrophic methanogenesis and subsequent methane oxidation, resulting in the relatively “heavy” isotopic signature of methane from Q. Danta.

The fact that methanogenesis is a relatively favorable bioenergetic process and that methanogens and ANME-1 were abundant at Spring 8 and Spring 9, even though the δ^13^C-CH_4_ in these samples is very enriched, might be explained by the hydrology of the system. Spring 8 and Spring 9 are located at the bottom of Murciélago watershed, where water flow paths from the recharge zone (~700 m a.s.l.) to the hyperalkaline springs outflow (78 m a.s.l.) are expected to be longer. Q. Danta is located in a steeper watershed but closer to the recharge zone, resulting in potentially shorter flow paths. In terms of water mean residence time (MRT), the expected longer MRT in Spring 8 and 9 might result in a stronger abiotic methane signature, by allowing abiogenic methane to accumulate, which in turn, may mask the biological methane signature. While in Q. Danta, having shorter water flow paths from the recharge zone to the spring outflow and consequently shorter MRT, biological methanogenesis likely impacts the bulk methane signal.

The hyperalkaline springs at SEO contain a greater abundance of Archaea and a higher proportion of methanogens than has been detected in any terrestrial serpentinizing system to date. The conditions at SEO appear to sustain a predominantly methanogenic ecosystem, where biological methanogenesis is sustained by acetoclastic and hydrogenotrophic processes, in concert with abiogenic methane formation. Methanogenic activities are likely to be facilitated by the dynamic flux of nutrients, including DIC, from surface water and their infiltration into serpentinizing groundwater during intense seasonal cycles of rainfall and recharge. However, in addition to methanogenic process, anaerobic methane oxidation may be superimposed and influence the resulting gas flux. These data provide new insight into methane cycle in a tropical serpentinizing environment, and context to the patterns in methane occurrence in terrestrial serpentinites in various hydrological and climatic settings.

## Author contributions

MC and KT contributed equally to this study. MC, RS, and MS designed the project and planned the field campaign. MC and RS were in charge of sample collection. MC and MS were in charge of geochemical characterization of the fluids, RS of isotopic analysis, KT and WB of sequence analysis, TM of the bioenergetics calculations. All authors contributed to the writing and editing of the manuscript.

## Funding

Metagenomic analysis was funded by the Census of Deep Life (CoDL) project PRJNA340462 to MC and RS. RS would like to thank the support of the Research Office of the National University of Costa Rica through grants SIA-0482-13, SIA-0378-14, and SIA-0101-14. Travel support, geochemical, and 16S rDNA sequence analyses were supported by MSU new faculty startup funds to MS. Postdoctoral salary support was provided to MC through a grant by the Deep Carbon Observatory (Alfred P. Sloan Foundation).

### Conflict of interest statement

The authors declare that the research was conducted in the absence of any commercial or financial relationships that could be construed as a potential conflict of interest.
