# Supplementary material for: Methane Dynamics in a Tropical Serpentinizing Environment: The Santa Elena Ophiolite, Costa Rica
Source: Front Microbiol. 2017 May 23;8:916. doi: 10.3389/fmicb.2017.00916 (PMC5440473; doi:10.3389/fmicb.2017.00916)
Supplement: Supplementary file 1 [file Table1.DOCX]

Supplementary Table S1**.** Metagenomic assembly statistics.

|  |  |  |  |  |  |  |  |  |
| --- | --- | --- | --- | --- | --- | --- | --- | --- |
|  | | | **Spring 9** | | **Murciélago Upstream** | | | |
| **Metagenome size (bp)** | | | 60,780,480 | | 45,476,317 | | | |
| **Number of contigs** | | | 25,100 | | 9,856 | | | |
| **Longest contig (bp)** | | | 95,356 | | 200,539 | | | |
| **Fold coverage** | | | 84 | | 150 | | | |
| **Number of ORFs** | | | 45,720 | | 23,943 | | | |
| **N50 (bp)** | | | 2,106 | | 2,921 | | | |
| **Reads Assembled (% of Total)** | | | 54.5 | | 54.2 | | | |
