# Supplementary material for: Methane Dynamics in a Tropical Serpentinizing Environment: The Santa Elena Ophiolite, Costa Rica
Source: Front Microbiol. 2017 May 23;8:916. doi: 10.3389/fmicb.2017.00916 (PMC5440473; doi:10.3389/fmicb.2017.00916)
Supplement: Supplementary file 2 [file Table2.DOCX]

Table S2. Quantification of methane-cycling genes searched for within the metagenomes (normalized reads per fragment). Data was normalized to the total number of fragments in the smaller metagenome. KEGG ID’s denoted by two asterisks (**) represents genes that were found in both metagenomes, while those denoted by one asterisk (*) were found only in the Spring 9 metagenome. No genes were found only in the Murciélago Upstream metagenome.

|  |  |  | **Normalized Reads per Fragment (RPF)** | |
| --- | --- | --- | --- | --- |
| **KEGG ID** | **Gene** | **Name** | **Spring 9** | **Murciélago Upstream** |
| K00123** | formate dehydrogenase | *fdoG, fdfH* | 1792.91 | 9445.79 |
| K00124** | formate dehydrogenase | *fdoH* | 1030.46 | 3903.61 |
| K00125* | formate dehydrogenase | E1.2.1.2B2 | 175.33 | 0.00 |
| K00126 | formate dehydrogenase | *fdsD* | 0.00 | 0.00 |
| K00127** | formate dehydrogenase | *fdoI* | 1145.03 | 5580.30 |
| K00200 | formylmethanofuran dehydrogenase | *fwdA, fmdA* | 8.62 | 0.00 |
| K00201 | formylmethanofuran dehydrogenase | *fwdB, fmdB* | 150.79 | 0.00 |
| K00202 | formylmethanofuran dehydrogenase | *fwdC, fmdC* | 0.00 | 0.00 |
| K00203 | formylmethanofuran dehydrogenase | *fwdD, fmdD* | 131.78 | 0.00 |
| K00204 | 4Fe-4S ferredoxin | *fwdH* | 0.00 | 0.00 |
| K00205* | 4Fe-4S ferredoxin | *fwdF, fmdF* | 152.09 | 0.00 |
| K11260* | 4Fe-4S ferredoxin | *fwdG* | 165.88 | 0.00 |
| K11261 | formylmethanofuran dehydrogenase | *fwdE, fmdE* | 0.00 | 0.00 |
| K00672* | formylmethanofuran--tetrahydromethanopterin N-formyltransferase | *ftr* | 18.40 | 0.00 |
| K01499* | methenyltetrahydromethanopterin cyclohydrolase | *mch* | 139.34 | 0.00 |
| K00319* | methylenetetrahydromethanopterin dehydrogenase | *mtd* | 129.80 | 0.00 |
| K13942 | 5,10-methenyltetrahydromethanopterin hydrogenase | *hmd* | 0.00 | 0.00 |
| K00440** | coenzyme F420 hydrogenase | *frhA* | 0.00 | 0.00 |
| K00441** | coenzyme F420 hydrogenase | *frhB* | 364.19 | 24.21 |
| K00442 | coenzyme F420 hydrogenase | *frhD* | 0.00 | 0.00 |
| K00443 | coenzyme F420 hydrogenase | *frhG* | 0.00 | 0.00 |
| K00320* | 5,10-methylenetetrahydromethanopterin reductase | *mer* | 178.31 | 0.00 |
| K00577* | tetrahydromethanopterin S-methyltransferase | *mtrA* | 121.23 | 0.00 |
| K00578* | tetrahydromethanopterin S-methyltransferase | *mtrB* | 103.07 | 0.00 |
| K00579* | tetrahydromethanopterin S-methyltransferase | *mtrC* | 114.32 | 0.00 |
| K00580* | tetrahydromethanopterin S-methyltransferase | *mtrD* | 125.78 | 0.00 |
| K00581* | tetrahydromethanopterin S-methyltransferase | *mtrE* | 148.24 | 0.00 |
| K00582 | tetrahydromethanopterin S-methyltransferase | *mtrF* | 0.00 | 0.00 |
| K00583* | tetrahydromethanopterin S-methyltransferase | *mtrG* | 223.51 | 0.00 |
| K00584* | tetrahydromethanopterin S-methyltransferase | *mtrH* | 153.48 | 0.00 |
| K00399* | methyl-coenzyme M reductase | *mcrA* | 152.15 | 0.00 |
| K00401* | methyl-coenzyme M reductase | *mcrB* | 154.33 | 0.00 |
| K00402* | methyl-coenzyme M reductase | *mcrG* | 46.90 | 0.00 |
| K03388* | heterodisulfide reductase | *hdrA* | 284.39 | 0.00 |
| K03389* | heterodisulfide reductase | *hdrB* | 11.06 | 0.00 |
| K03390* | heterodisulfide reductase | *hdrC* | 174.66 | 0.00 |
| K00925** | acetate kinase | *ackA* | 569.43 | 222.76 |
| K00625** | phosphate acetyltransferase | *pta* | 3372.74 | 452.66 |
| K13788 | phosphate acetyltransferase | *pta* | 0.00 | 0.00 |
| K01895** | AMP-forming acetyl-CoA synthetase | *acs* | 4706.60 | 9737.09 |
| K00192* | acetyl-CoA decarbonylase/synthase | *cdhA* | 140.33 | 0.00 |
| K00193* | acetyl-CoA decarbonylase/synthase | *cdhC* | 19.96 | 0.00 |
| K00194* | acetyl-CoA decarbonylase/synthase | *cdhD, acsD* | 121.80 | 0.00 |
| K00195* | acetyl-CoA decarbonylase/synthase | *cdhB* | 337.54 | 0.00 |
| K00196 | carbon-monoxide dehydrogenase | *cooF* | 0.00 | 0.00 |
| K00197* | acetyl-CoA decarbonylase/synthase | *cdhE, acsC* | 173.17 | 0.00 |
| K00198 | carbon-monoxide dehydrogenase | *cooS, acsA* | 0.00 | 0.00 |
| K14080 | [methyl-Co(III) methanol-specific corrinoid protein]:coenzyme M methyltransferase | *mtaA* | 0.00 | 0.00 |
| K04480 | methanol---5-hydroxybenzimidazolylcobamide Co-methyltransferase | *mtaB* | 0.00 | 0.00 |
| K14081 | methanol corrinoid protein | *mtaC* | 0.00 | 0.00 |
| K14082 | [methyl-Co(III) methylamine-specific corrinoid protein]:coenzyme M methyltransferase | *mtbA* | 0.00 | 0.00 |
| K14083 | trimethylamine---corrinoid protein Co-methyltransferase | *mttB* | 0.00 | 0.00 |
| K14084 | trimethylamine corrinoid protein | *mttC* | 0.00 | 0.00 |
| K16176 | methylamine---corrinoid protein Co-methyltransferase | *mtmB* | 0.00 | 0.00 |
| K16177 | monomethylamine corrinoid protein | *mtmC* | 0.00 | 0.00 |
| K16178 | dimethylamine---corrinoid protein Co-methyltransferase | *mtbB* | 0.00 | 0.00 |
| K16179 | dimethylamine corrinoid protein | *mtbC* | 0.00 | 0.00 |
| K16157 | methane mooxygenase | *mmoX* | 0.00 | 0.00 |
| K16158 | methane mooxygenase | *mmoY* | 0.00 | 0.00 |
| K16159 | methane mooxygenase | *mmoZ* | 0.00 | 0.00 |
| K16161 | methane mooxygenase | *mmoC* | 0.00 | 0.00 |
| K10944 | methane/ammonia monooxygenase | *pmoA, amoA* | 0.00 | 0.00 |
| K00368 | nitrite reductase (NO-forming) | *nirK* | 0.00 | 0.00 |
| K15864 | nitrite reductase (NO-forming) / hydroxylamine reductase | *nirS* | 0.00 | 0.00 |
| K14028 | methanol dehydrogenase | *mdh1, mxaF* | 0.00 | 0.00 |
