# Supplementary material for: Methane Dynamics in a Tropical Serpentinizing Environment: The Santa Elena Ophiolite, Costa Rica
Source: Front Microbiol. 2017 May 23;8:916. doi: 10.3389/fmicb.2017.00916 (PMC5440473; doi:10.3389/fmicb.2017.00916)
Supplement: Supplementary file 3 [file Table3.DOCX]

Table S3. Free energy yield for methagenomic reactions

|  | **kJ/mol reaction** | **kJ/L fluid** | **Limiting Reactant** |
| --- | --- | --- | --- |
| ***Hydrogenotrophic methanogenesis (4H_2_ + CO_2_ -> CH_4_ + 2H_2_O)*** | | | |
| Q.Danta | -54.41 | -5.20 x 10^-4^ | hydrogen |
| Spring 9 | -56.53 | -7.51 x 10^-4^ | hydrogen |
| Spring 8 | -40.91 | -1.11 x 10^-4^ | hydrogen |
| Murciélago Upstream | -48.04 | -9.05 x 10^-6^ | hydrogen |
| Q.Danta Upstream | -85.60 | -1.96 x 10^-4^ | hydrogen |
| R.Calera | -95.04 | -5.62 x 10^-4^ | hydrogen |
| P. Murciélago | -69.55 | -1.67 x 10^-5^ | hydrogen |
| P. Nuevo | -61.48 | -1.22 x 10^-5^ | hydrogen |
| P. Aguas Calientes | -70.70 | -1.67 x 10^-5^ | hydrogen |
| ***Acetoclastic methanogenesis (CH_3_COO^-^ + H^+^ -> CH_4_ + CO_2_);* Acetate = 10% of DOC** | | | |
| Q.Danta | -31.65 | -1.77 x 10^-4^ | acetate |
| Spring 9 | -28.90 | -1.19 x 10^-3^ | acetate |
| Spring 8 | -30.44 | -2.23 x 10^-3^ | acetate |
| Murciélago Upstream | -32.07 | -2.15 x 10^-3^ | acetate |
| Q.Danta Upstream | -41.82 | -4.31 x 10^-3^ | acetate |
| R.Calera | -40.56 | -2.41 x 10^-3^ | acetate |
| P. Murciélago | -36.35 | -4.23 x 10^-4^ | acetate |
| P. Nuevo | -38.60 | -7.80 x 10^-4^ | acetate |
| P. Aguas Calientes | -40.06 | -1.04 x 10^-3^ | acetate |
| ***Acetoclastic methanogenesis (CH_3_COO^-^ + H^+^ -> CH_4_ + CO_2_);* Acetate = 1% of DOC** | | | |
| Q.Danta | -25.95 | -1.45 x 10^-4^ | acetate |
| Spring 9 | -23.19 | -9.55 x 10^-4^ | acetate |
| Spring 8 | -24.73 | -1.81 x 10^-3^ | acetate |
| Murciélago Upstream | -26.37 | -1.77 x 10^-3^ | acetate |
| Q.Danta Upstream | -36.12 | -3.72 x 10^-3^ | acetate |
| R.Calera | -34.85 | -2.07 x 10^-3^ | acetate |
| P. Murciélago | -30.64 | -3.57 x 10^-4^ | acetate |
| P. Nuevo | -32.89 | -6.65 x 10^-4^ | acetate |
| P. Aguas Calientes | -34.35 | -8.88 x 10^-4^ | acetate |
