# Supplementary material for: Methane Dynamics in a Tropical Serpentinizing Environment: The Santa Elena Ophiolite, Costa Rica
Source: Front Microbiol. 2017 May 23;8:916. doi: 10.3389/fmicb.2017.00916 (PMC5440473; doi:10.3389/fmicb.2017.00916)
Supplement: Supplementary file 4 [file Table4.DOCX]

Table S4. Free energy yield for methanotrophic reactions

|  | **kJ/mol reaction** | **kJ/L fluid** | **Limiting Reactant** |
| --- | --- | --- | --- |
| ***Anaerobic Methane Oxidation with Sulfate (CH_4_ + SO_4_^-2^ + H^+^ -> CO_2_ + HS^-^ + 2 H_2_O)*** | | | |
| Q.Danta | -47.64 | -1.64 x 10^-4^ | sulfate |
| Spring 9 | -49.06 | -1.69 x 10^-4^ | sulfate |
| Spring 8 | -48.95 | -1.68 x 10^-4^ | sulfate |
| Murciélago Upstream | -33.65 | -4.82 x 10^-4^ | methane |
| Q.Danta Upstream | -24.50 | -7.98 x 10^-6^ | methane |
| R. Calera | -22.53 | -5.75 x 10^-6^ | methane |
| P. Murciélago | -19.08 | -4.86 x 10^-6^ | methane |
| P. Nuevo | -20.94 | -6.50 x 10^-6^ | methane |
| P. Aguas Calientes | -25.24 | -4.84 x 10^-6^ | methane |
| ***Anaerobic Methane Oxidation with Nitrate (CH_4_ + 4NO_3_^-^ -> CO_2_ + 4NO_2_^-^ + 2H_2_O)*** | | | |
| Q.Danta | -585.49 | -1.09 x 10^-4^ | nitrate |
| Spring 9 | -570.96 | -2.57 x 10^-5^ | nitrate |
| Spring 8 | -577.77 | -4.28 x 10^-5^ | nitrate |
| Murciélago Upstream | -534.22 | -3.69 x 10^-5^ | nitrate |
| Q.Danta Upstream | -539.51 | -1.76 x 10^-4^ | methane |
| R. Calera | -542.04 | -1.38 x 10^-4^ | methane |
| P. Murciélago | -574.05 | -1.46 x 10^-4^ | methane |
| P. Nuevo | -525.20 | -1.63 x 10^-4^ | methane |
| P. Aguas Calientes | -566.07 | -1.08 x 10^-4^ | methane |
| ***Aerobic Methanotrophy (CH_4_ + 2O_2_ -> CO_2_ + 2H_2_O);* O_2_= 0.1% saturation** | | | |
| Q.Danta | -818.09 | -4.09 x 10^-5^ | oxygen |
| Spring 9 | -819.22 | -4.10 x 10^-5^ | oxygen |
| Spring 8 | -819.10 | -4.10 x 10^-5^ | oxygen |
| Murciélago Upstream | -785.53 | -3.93 x 10^-5^ | oxygen |
| Q.Danta Upstream | -772.73 | -3.86 x 10^-5^ | oxygen |
| R. Calera | -772.69 | -3.86 x 10^-5^ | oxygen |
| P. Murciélago | -766.42 | -3.83 x 10^-5^ | oxygen |
| P. Nuevo | -772.61 | -3.86 x 10^-5^ | oxygen |
| P. Aguas Calientes | -765.14 | -3.83 x 10^-5^ | oxygen |
| ***Aerobic Methanotrophy (CH_4_ + 2O_2_ -> CO_2_ + 2H_2_O);* O_2_= 1.0% saturation** | | | |
| Q.Danta | -829.50 | -4.15 x 10^-4^ | oxygen |
| Spring 9 | -830.63 | -4.15 x 10^-4^ | oxygen |
| Spring 8 | -830.51 | -4.15 x 10^-4^ | oxygen |
| Murciélago Upstream | -796.94 | -3.98 x 10^-4^ | oxygen |
| Q.Danta Upstream | -784.14 | -2.52 x 10^-4^ | methane |
| R. Calera | -784.11 | -1.97 x 10^-4^ | methane |
| P. Murciélago | -777.83 | -1.95 x 10^-4^ | methane |
| P. Nuevo | -784.02 | -2.40 x 10^-4^ | methane |
| P. Aguas Calientes | -776.55 | -1.47 x 10^-4^ | methane |
