# Supplementary material for: Methane Dynamics in a Tropical Serpentinizing Environment: The Santa Elena Ophiolite, Costa Rica
Source: Front Microbiol. 2017 May 23;8:916. doi: 10.3389/fmicb.2017.00916 (PMC5440473; doi:10.3389/fmicb.2017.00916)
Supplement: Supplementary file 5 [file Table5.DOCX]

Table S5. Geochemical and isotopic data for samples collected from Santa Elena Ophiolite (Average data presented in Table 1)^1^

|  | **Geochemical Parameters** | | | | | | | | **Gaseous Geochemistry and isotopic composition** | | | | | | |
| --- | --- | --- | --- | --- | --- | --- | --- | --- | --- | --- | --- | --- | --- | --- | --- |
| **Sample Name** | **DOC (µM)** | **TDN (µM)** | **NOx (µM)** | **NO_2_ (µM)** | **NH_4_^+^ (µM)** | **DON (µM)** | **PO_4_^3-^ (µM)** | **TDP (µM)** | **CH_4_ (µM)** | **δ^13^C-CH_4_ (‰)** | **δ^13^C-CH_4_ SD (‰)^2^** | **H_2_ (µM)** | **DIC (µM)** | **δ^13^C-CO_2_ (‰)** | **δ^13^C-CO_2_ SD (‰)** |
| Q.Danta | 7.9 | bdl | 0.3 | 0.03 | 1.57 | bdl | 0.09 | 0.17 | 145 | -43.86 | 0.22 | 45.62 | 119 | -18.66 | 0.81 |
| Q.Danta | 3.5 | 0.3 | 0.2 | 0.01 | 1.3 | bdl | 0.05 | 0.08 | 145 | -44.07 | 0.19 | 15.53 | 85 | -22.68 | 1.13 |
| Q.Danta | 5.4 | 0.4 | 1.8 | 0.01 | 1.16 | bdl | 0.09 | 0.08 | 145 | -44.18 | 0.18 | 53.6 | 175 | -18.85 | 0.65 |
| Q.Danta Upstream | 101 | 6.6 | 2.7 | 0.07 | 0.69 | 3.2 | 1.52 | 0.17 | 0.36 | -36.4 | 18.8 | 8.33 | 632 | -17.04 | 0.65 |
| Q.Danta Upstream | 105 | 7 | 2.5 | 0.07 | 0.48 | 4 | 1.56 | 0.27 | 0.29 | -37.67 | 18.35 | 18.22 | 608 | -17.86 | 0.57 |
| Q.Danta Upstream | 103 | 6.1 | 2.4 | 0.07 | 0.55 | 3.2 | 1.6 | 0.13 | 0.33 | -37.97 | 16.02 | 0.95 | 552 | -18.78 | 0.77 |
| Spring 8 | 73.1 | 14.8 | 0.3 | 0.01 | 1.77 | 12.7 | 0.22 | 0.13 | 905 | -1.71 | 0.3 | 4.27 | 222 | -17.99 | 0.89 |
| Spring 8 | 70.4 | 13.4 | 0.3 | 0.01 | 1.77 | 11.3 | 0.22 | 0.13 | 889 | -1.54 | 0.36 | 6.48 | 315 | -15.94 | 0.44 |
| Spring 8 | 76 | 14.5 | 0.3 | 0.03 | 1.64 | 12.5 | 0.22 | 0.13 | 818 | 0.51 | 0.31 | 21.8 | 144 | -19.79 | 1.04 |
| Spring 9 | 45.3 | 2.4 | 0.4 | 0.01 | 0.75 | 1.3 | 0.31 | 0.03 | 944 | -2.51 | 0.48 | 36.19 | 293 | -17.35 | 0.66 |
| Spring 9 | 36.8 | 0.9 | 0.2 | 0.03 | 0.82 | bdl | 0.22 | 0.08 | 903 | -1.86 | 0.45 | 34.8 | 237 | -22.72 | 1.91 |
| Spring 9 | 41.4 | 1.9 | 0.1 | 0.03 | 0.96 | 0.9 | 0.27 | 0.08 | 890 | -2.22 | 0.24 | 88.35 | 233 | -22.35 | 2.25 |
| Murciélago Upstream | 67.3 | 1.8 | 0.4 | 0.05 | 0.14 | 1.3 | 1.6 | 0.08 | 14 | 1.77 | 0.63 | 0.8 | 506 | -19.98 | 0.68 |
| Murciélago Upstream | 67.6 | 1.9 | 0.3 | 0.07 | 0.14 | 1.4 | 1.52 | 0.41 | 15 | 1.2 | 0.65 | 0.69 | 529 | -20.08 | 0.76 |
| Murciélago Upstream | 66.3 | 1.5 | 0.3 | 0.05 | 0.14 | 1.1 | 1.56 | 0.13 | 14 | 0.98 | 0.62 | 0.78 | 566 | -19.43 | 0.66 |
| R.Calera | 54.5 | 2.6 | 1.7 | 0.03 | 0.07 | 0.8 | 1.9 | 0.22 | 0.42 | -22 | 19.23 | 69.22 | 720 | -18.76 | 0.5 |
| R.Calera | 63.7 | 2.6 | 1.9 | 0.03 | 0.14 | 0.6 | 1.77 | 0.13 | 0.22 | -22.83 | 23.22 | 0.39 | 635 | -18.69 | 0.61 |
| R.Calera | 60.3 | 2.6 | 1.9 | 0.05 | 0.07 | 0.6 | 1.9 | 0.13 | 0.13 | -9.67 | 35.32 | 1.36 | 635 | -18.46 | 0.63 |
| P. Murciélago | 8.2 | 61.3 | 58.5 | 0.01 | 2.11 | 0.7 | 2.42 | 0.41 | 0.32 | 3.07 | 40.7 | 0.62 | 723 | -19.63 | 0.48 |
| P. Murciélago | 12.1 | 62.2 | 57.8 | 0.03 | 1.84 | 2.6 | 2.85 | 0.41 | 0.24 | 5.59 | 52.66 | 1.05 | 792 | -19.51 | 0.48 |
| P. Murciélago | 14.6 | 63.7 | 58.7 | 0.03 | 2.38 | 2.6 | 2.68 | 0.41 | 0.2 | 9.44 | 66.39 | 1.21 | 729 | -19.24 | 0.59 |
| P. Nuevo | 12.6 | 3.6 | 0.2 | 0.03 | 4.08 | bdl | 1.6 | 0.55 | 0.11 | 0.71 | 57.07 | 0.65 | 437 | -18.18 | 0.84 |
| P. Nuevo | 19.6 | 3.6 | 0.3 | 0.01 | 3.95 | bdl | 1.64 | 0.74 | 0.4 | -22.13 | 20.35 | 1.08 | 406 | -18.72 | 0.73 |
| P. Nuevo | 28.4 | 3.4 | 0.1 | 0.03 | 4.36 | bdl | 1.82 | 0.55 | 0.43 | -29.57 | 14.85 | 0.65 | 410 | -18.84 | 0.65 |
| P. Aguas Calientes | 26 | 38.3 | 36.7 | 0.03 | 0.89 | 0.7 | 2.55 | 1.06 | 0.27 | -11.51 | 22.79 | 1.78 | 632 | -18.89 | 0.65 |
| P. Aguas Calientes | 28.3 | 40.4 | 37.2 | 0.03 | 1.64 | 1.5 | 2.77 | 1.11 | 0.17 | 2.52 | 35.59 | 0.68 | 687 | -18.59 | 0.52 |
| P. Aguas Calientes | 23.3 | 38.9 | 36.6 | 0.03 | 1.84 | 0.5 | 2.89 | 1.11 | 0.14 | 16.91 | 40.67 | 0.39 | 614 | -18.68 | 0.57 |

SD: standard deviation; bdl: below detection limit

^1^Analyses were done as contracted services at the Joye Biogeochemistry Laboratory (University of Georgia, GA, USA; <http://www.joyeresearchgroup.uga.edu/>). The analytical methods used were: combustion for DOC and TDN, chemiluminescence for NOx, spectrophotometry for NO_2_, NH_4_^+^, PO_4_^3-^ and TDP, gas chromatography for H_2_, and cavity ringdown spectrometry for CH_4_, DIC and their respective isotopes. DON was calculated from TDN and DIN values.

^2^Samples with methane concentration below 0.3 µM were within the limit of the detection and resulted in δ^13^C-CH_4_ signature with an elevated standard deviation (16-66 ‰)
